# Supplementary material for: Uveal Melanoma Patients Have a Distinct Metabolic Phenotype in Peripheral Blood
Source: Int J Mol Sci. 2023 Mar 7;24(6):5077. doi: 10.3390/ijms24065077 (PMC10049075; doi:10.3390/ijms24065077)
Supplement: Supplementary file 1 [file ijms-24-05077-s001.zip › ijms-2239025-supplementary-word.pdf]

International Journal of
*Molecular Sciences*

MDPI

*Supplementary material*

# Uveal melanoma patients have a distinct metabolic phenotype in peripheral blood

**Daniël P. de Bruyn[1,2,3]†, Michiel Bongaerts[2]†, Ramon Bonte[2], Jolanda Vaarwater[1], Magda A. Meester-Smoor[1], Robert M. Verdijk[4,5,6], Dion Paridaens[1,4], Nicole C. Naus[1,3], Annelies de Klein[2,3], George J.G. Ruijter[2], Emine Kiliç[1,3]‡ & Erwin Brosens[2,3]‡*, on behalf of the Rotterdam Ocular Melanoma Study Group (ROMS)**

## Table of contents

Figure S1. Sample numbers in discovery, replication and merged datasets, 10-time bootstrap and leave-one-out cross-validation.

Figure S2. Quality control characteristics by internal standards in discovery set.

Figure S3. Quality control characteristics by internals standards in replication set.

Figure S4. Assessment of Metchalizer normalization in the merged dataset.

Figure S5. Assessment of technical and biological variation by using replicates in the discovery cohort and merged dataset.

Figure S6. P-value distribution of differentially abundant features in UM versus controls.

Figure S7. Quality control by dimensionality reduction analyses in the positive ion mode.

Figure S8. Quality control by dimensionality reduction analyses in the negative ion mode.

Figure S9. Repeated leave-one-out cross-validation on random forest classifier.

Figure S10. Receiver operating characteristic of the repeated leave-one-out cross-validation of the random forest classifiers.

Figure S11. Correlation of m/z features with tumor size and differential abundance between UM patients and controls.

Figure S12. Pathway analysis of top-10 affected pathways in UM patients compared to controls.

Table S1. Characteristics of controls.

Table S2. Patient and sample characteristics of the technical and biological replication sets.

Table S3. Differentially abundant annotated metabolites between UM patients and controls, differentially abundant unannotated features in positive ion mode, and differentially abundant unannotated features in negative ion mode

Supplementary materials references.

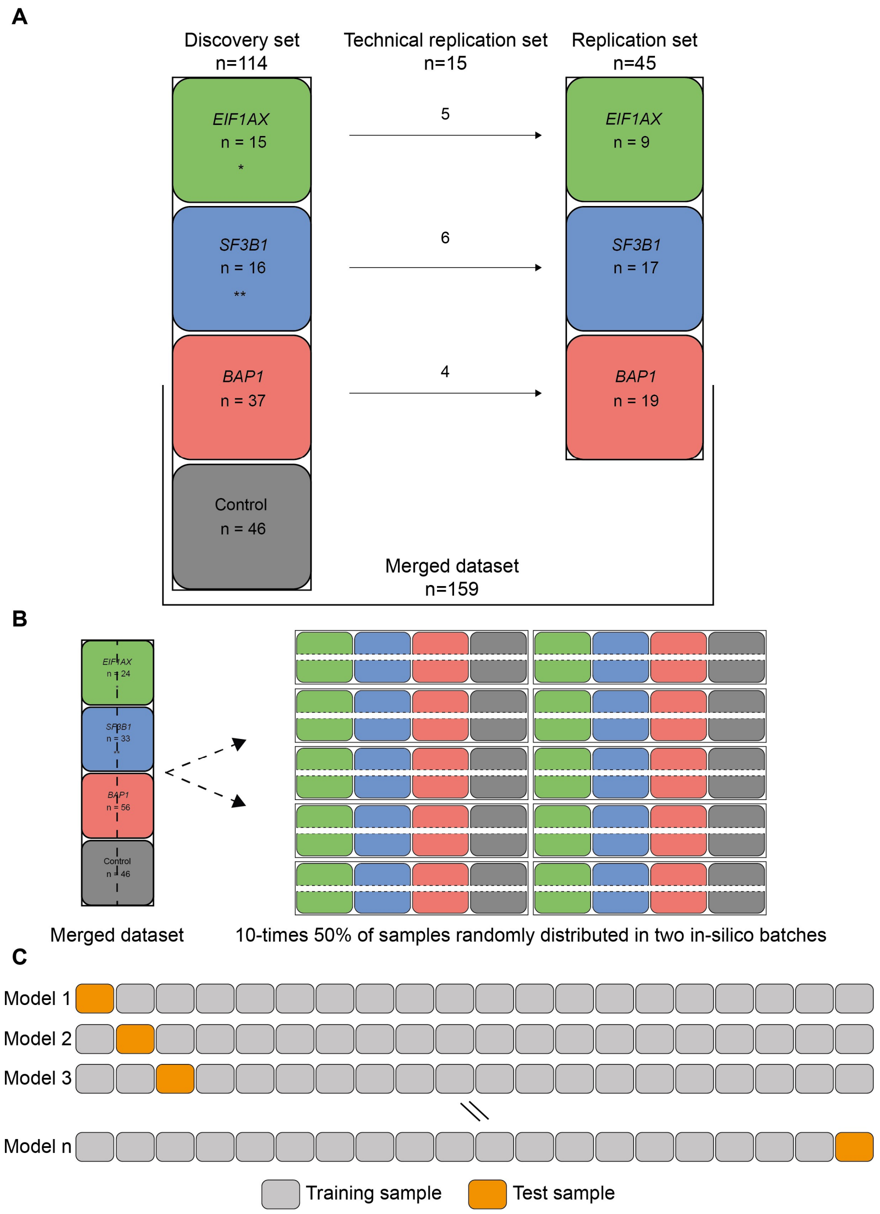


**Figure S1. Sample inclusion in discovery, replication and merged datasets, 10-time bootstrap and leave-one-out cross-validation**

159 participants enrolled in our study. Our discovery set comprised of 15 patients harboring an *EIF1AX*-mutated tumor, 16 patients harboring an *SF3B1*-mutated tumor and 37 patients harboring a *BAP1*-mutated tumor. Additionally, 46 control-participants were included in the discovery set. The replication set included 9 patients harboring an *EIF1AX*-mutated tumor, 17 patients harboring an *SF3B1*-mutated tumor and 19 patients harboring a *BAP1*-mutated tumor. To assess batch effects in the merged dataset (discovery and replication set combined), 15 samples were used as a technical replication set (see Figure S5). These technical replicates were used in the discovery cohort for exploring differential features (and subsequent metabolites). Afterwards, in the replication cohort, these samples were only used for quality control evaluation. Two patients harboring an *SF3B1*-mutated tumor and one patient harboring an *EIF1AX*-mutated tumor had an additional plasma sample, retrieved at the same time-point, included in the replication set as biological replicates to assess similarity in the metabolite profiles (**A**). To assess the reproducibility of using metabolite abundancies for random forest classifiers (RFC) and leave-one-out cross-validation (LOOCV), we repeated this ten times on samples that were randomly picked from the merged dataset and then evenly distributed in two in-silico batches in the respective groups (**B**). The LOOCV procedure entails using all-but-one samples to train a model and using the left-out sample to test the models' performance. This procedure is performed for all samples and produces performance metrics for all samples and equivalent models (**C**).

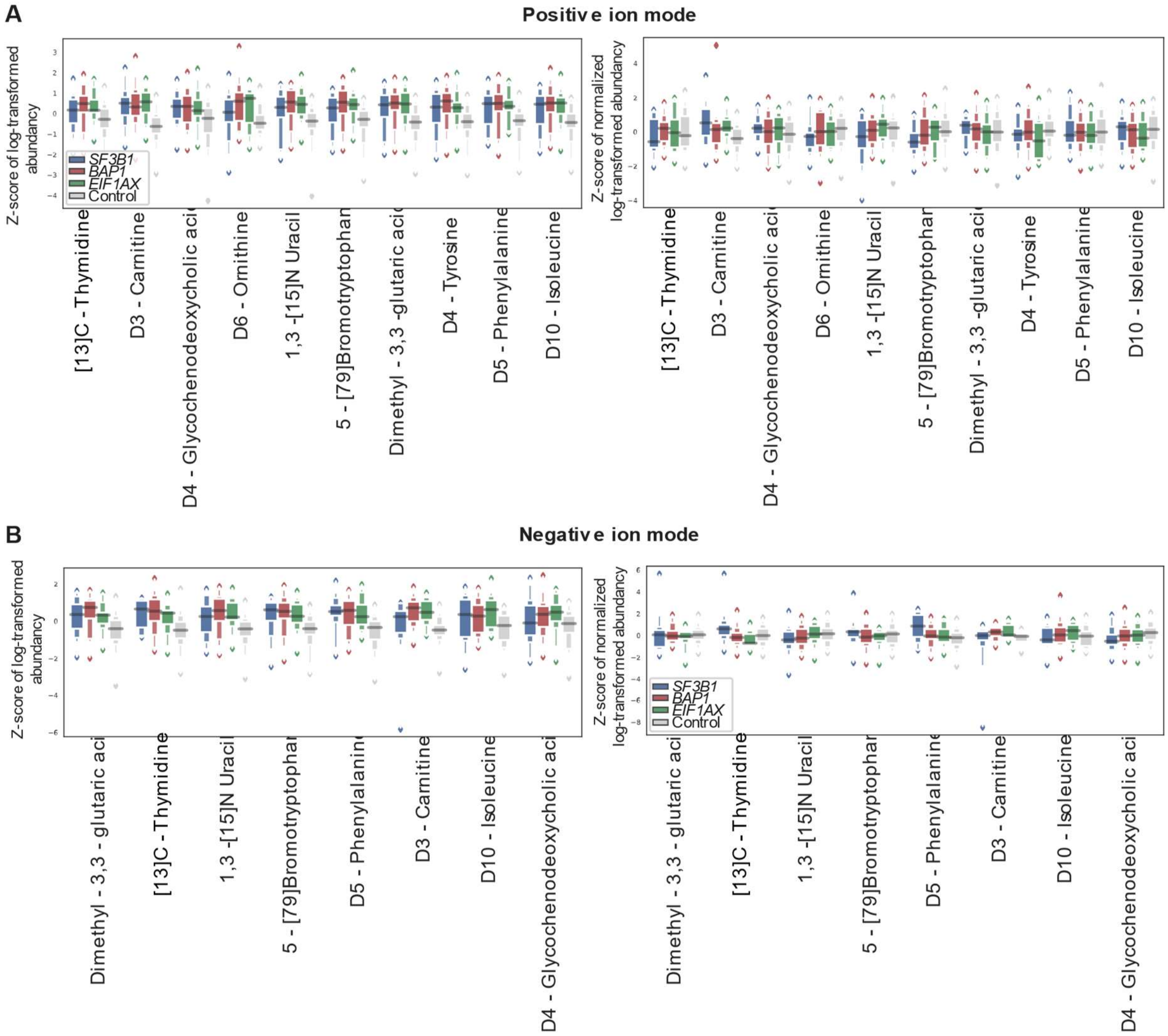


**Figure S2. Quality control characteristics by internal standards in discovery set.**

To eliminate technical variances, internal standards were added to each sample. These internal standards were used to normalize feature abundancies (see methods). For some groups we observe overall abundancy differences for the internal standards. Normalization reduced these group differences when looking at the normalized internal standards. Boxplots show z-scores of log-transformed abundancies of the internal standards in the discovery set in the positive ion mode (**A**) and negative ion mode (**B**); on the left are unnormalized abundancies and the right side shows normalized abundancies.

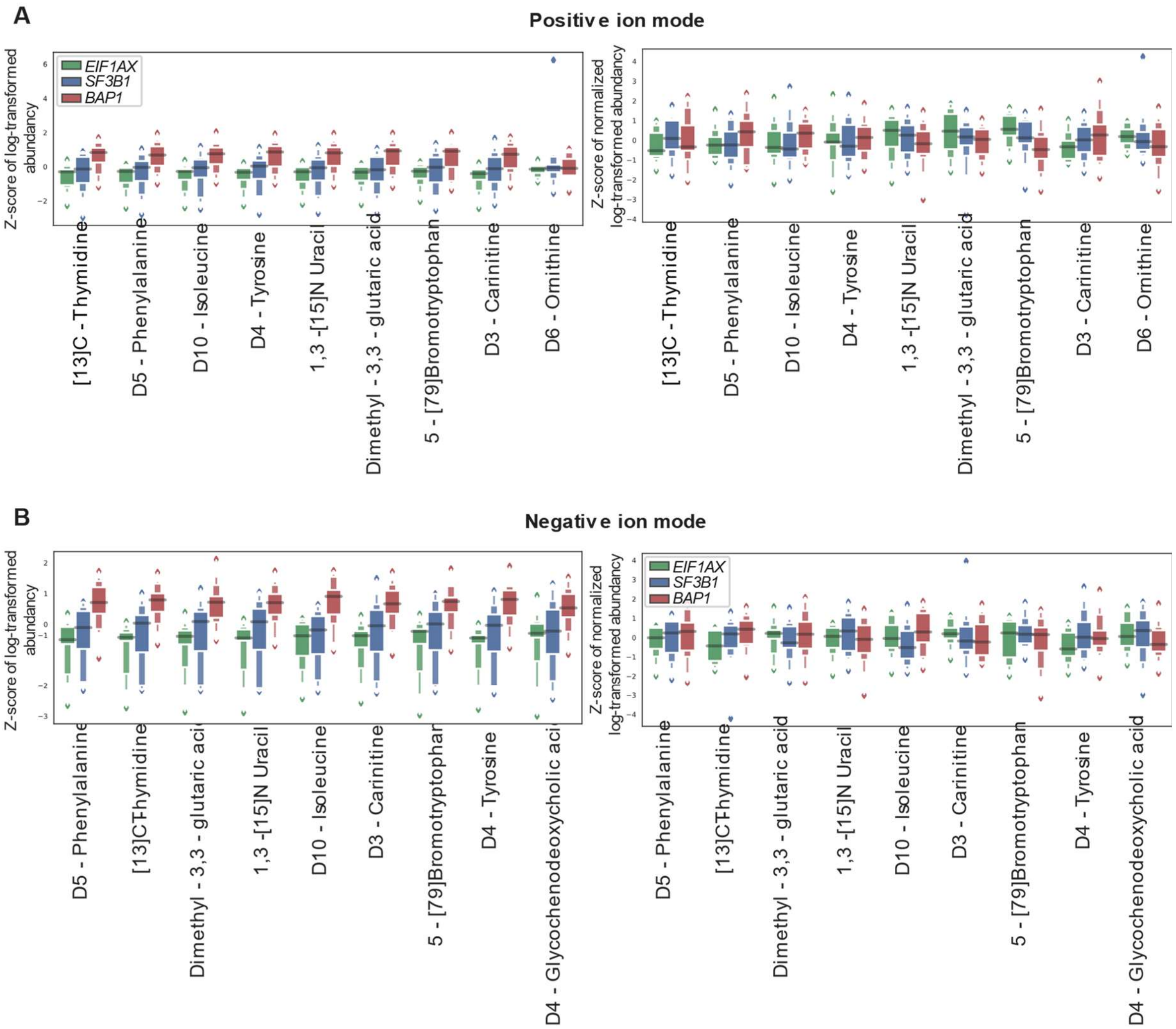


**Figure S3. Quality control characteristics by internals standards in replication set.**

To eliminate technical variances in the replication set, internal standards were used to normalize feature abundancies. After normalization, technical differences in metabolite abundancy were reduced in the positive ion mode (**A**) and negative ion mode (**B**).

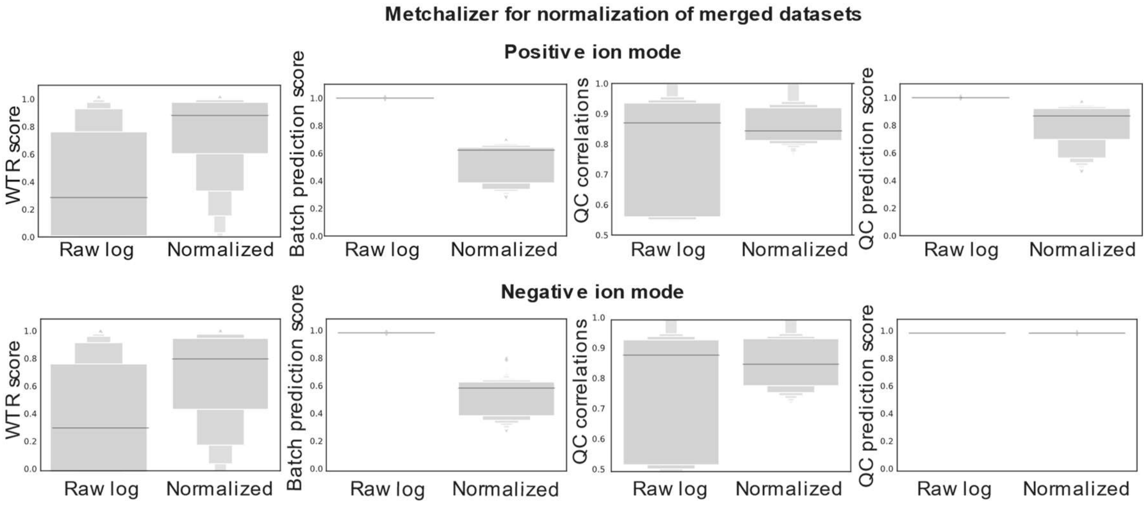


**Figure S4. Assessment of Metchalizer normalization in the merged dataset.**

The four metrics Within variance Total variance Ratio (WTR) scores, Batch prediction scores, quality control (QC) correlations and QC prediction scores are determined for both the log-transformed raw data and the Metchalizer normalized data. Technical replicates of the same sample were included in the replication and discovery set. Additionally, technical replicates were included in each individual dataset. Assessment of these technical replicates indicate improved normalization as they are associated with increased WTR scores, QC correlations and QC predictions scores, and with reduced batch prediction scores [1]. These metrics are determined for both ion modes.

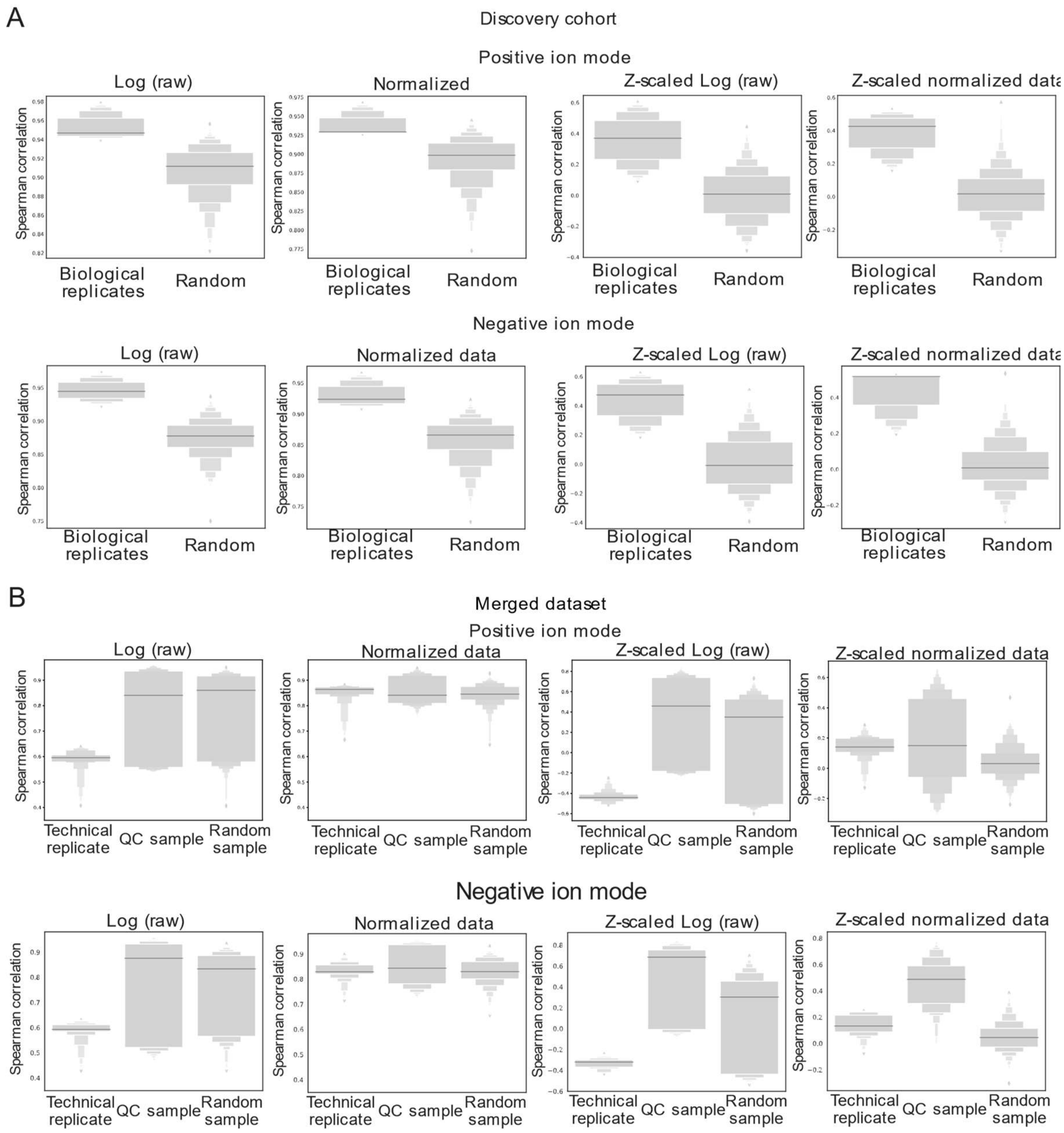


**Figure S5. Assessment of technical and biological variation by using replicates in the discovery cohort and merged dataset.**

The Spearman correlation between two samples was determined using all features for samples in the discovery cohort and merged dataset, where abundancies were either log-transformed raw abundancies or normalized abundancies. In the discovery cohort, these correlations were determined between biological replicates (two different UM-patient plasma samples retrieved at one time-point) or between a random selection of samples (**A**). For the latter we expect, and observe, lower correlation coefficients than the biological replicates. Furthermore, normalized abundancies should ideally further improve the correlation between the latter. In the merged dataset, correlations were determined between technical replicates (a UM-patient sample being measured in each of the two batches), between the quality control (QC) samples (same sample measured multiple times per batch and in both batches) and between a random selection of samples (**B**). The Spearman correlation of normalized feature abundancies of biological and technical replicates (both UM-patient samples and QC-samples) showed better correlation than randomly chosen samples.

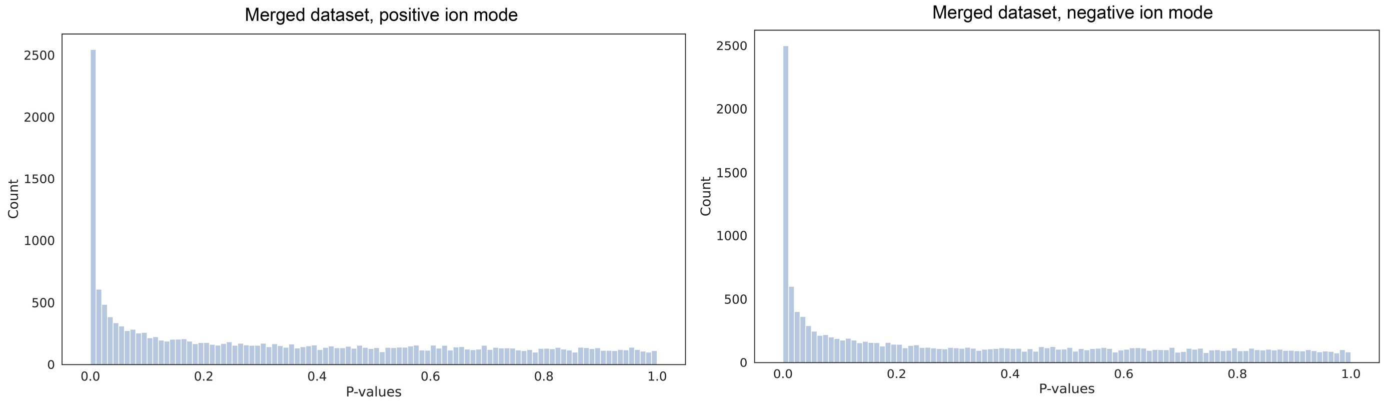


**Figure S6. P-value distribution of differentially abundant features in UM versus controls.**

The distribution of p-values obtained from the t-test by comparing feature abundancies between UM and controls. This distribution shows to be uniformly distributed except for p-values in the left tail for the merged dataset in the positive and negative ion modes, respectively. This shows that most features are not differentially abundant between UM patients and controls and that a subset of features are differentially abundant between UM patients and controls, which is a sign of good quality data.

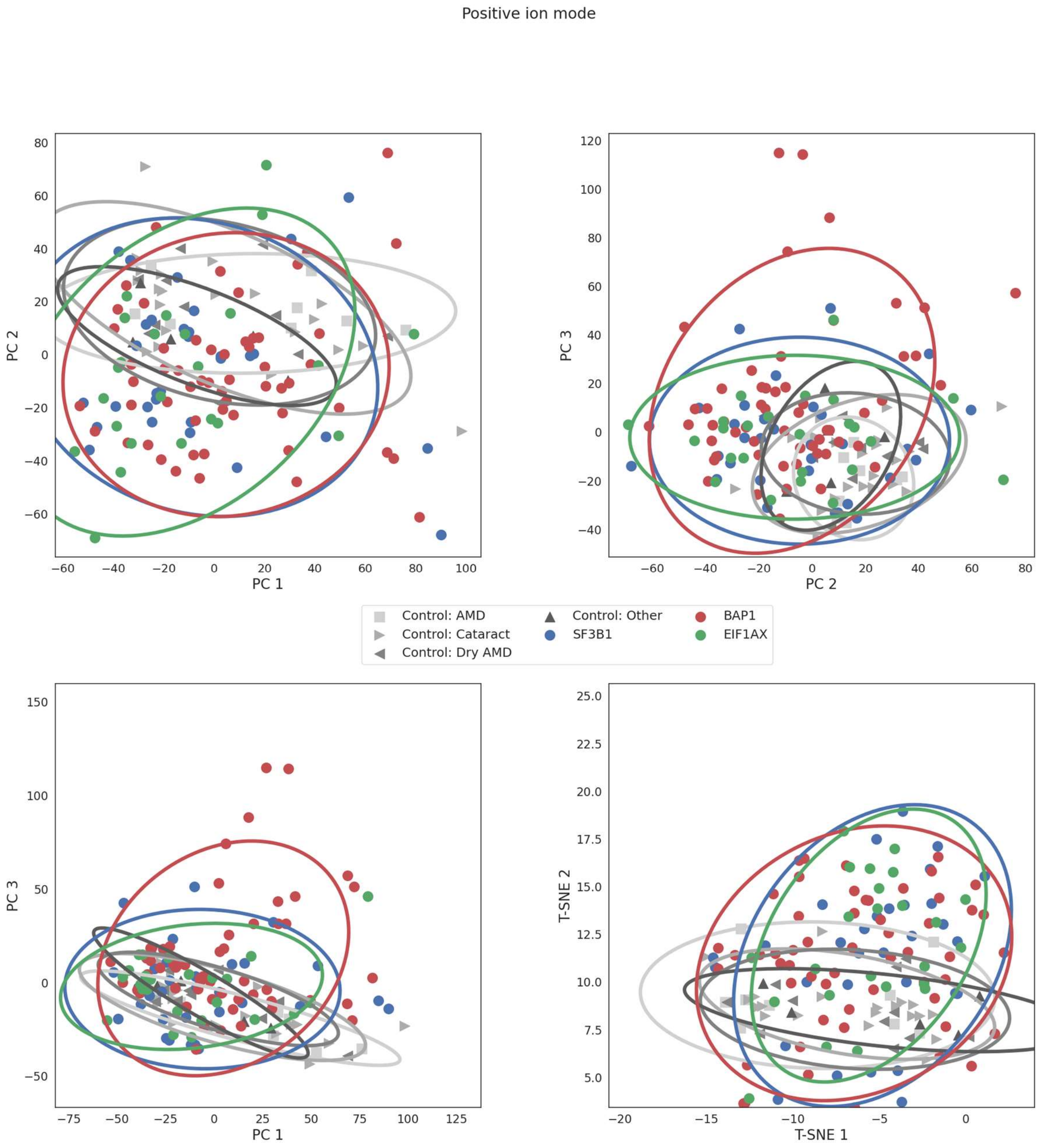


**Figure S7. Quality control by dimensionality reduction analyses in the positive ion mode.**

Unsupervised clustering by principal component analyses (PCA) and t-distributed Stochastic Neighbor Embedding (t-SNE) on all normalized feature abundancies show no deviations in three dimensions of the PCA and in two dimensions of the t-SNE (normalized) feature abundancies between plasma samples of patients with *BAP1*, *SF3B1* or *EIF1AX* and control-participants in the whole cohort consisting of the discovery and replication sets (i.e., the merged dataset) in the positive ion mode. The control participants are marked for their co-occurring diseases (consisting of AMD, cataract and others (Table S1) and no separation based on metabolite feature abundancies is observed.

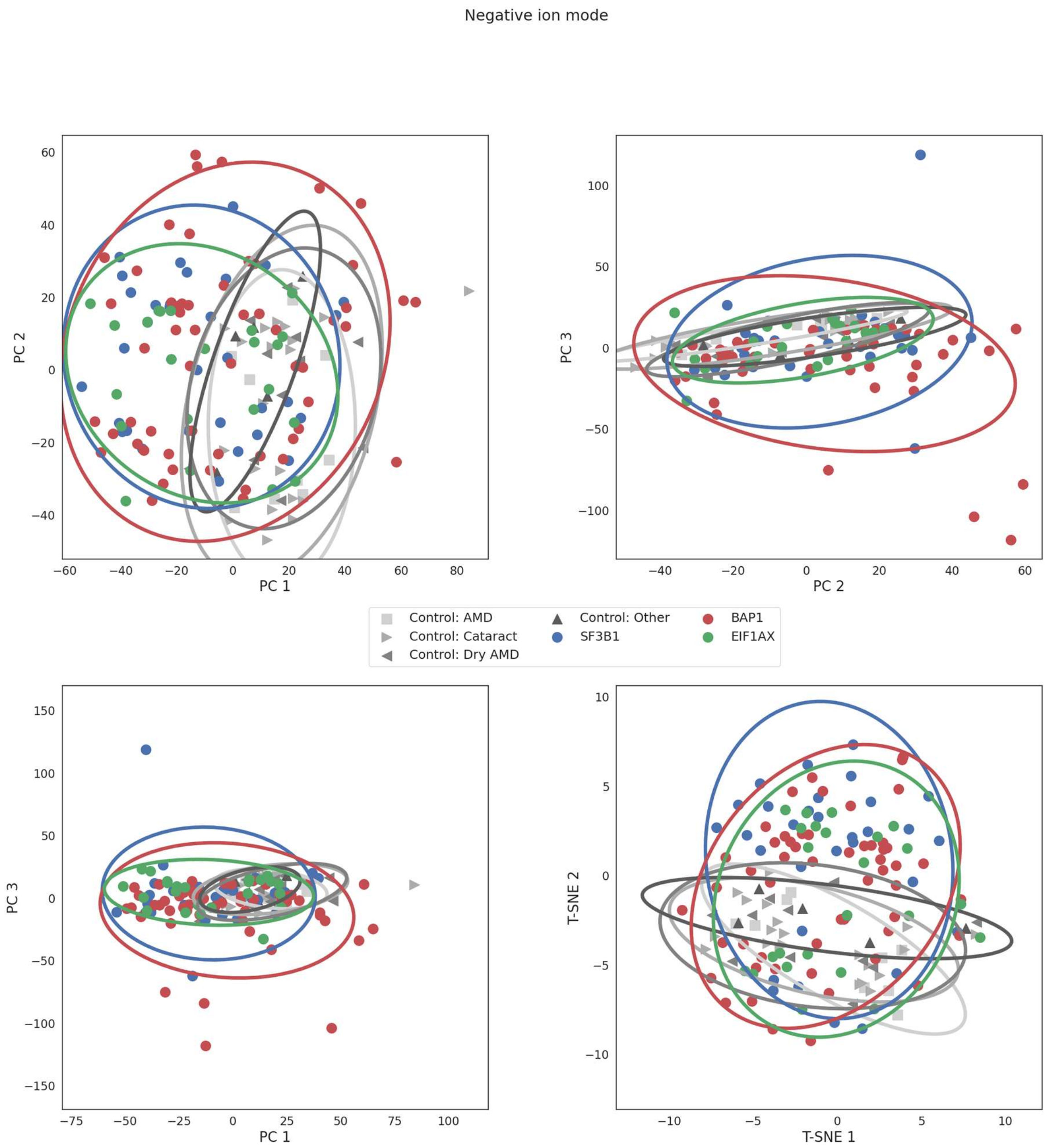


**Figure S8. Quality control by dimensionality reduction analyses in the negative ion mode.**

Unsupervised clustering by principal component analyses (PCA) and t-distributed Stochastic Neighbor Embedding (t-SNE) on all normalized feature abundancies show no deviations in three dimensions of the PCA and in two dimensions of the t-SNE (normalized) feature abundancies between plasma samples of patients with *BAP1*, *SF3B1* or *EIF1AX* and control-participants in the whole cohort consisting of the discovery and replication sets (i.e., the merged dataset) in the negative ion mode. The control participants are marked for their co-occurring diseases (consisting of AMD, cataract and others (Table S1) and no separation based on metabolite feature abundancies is observed.

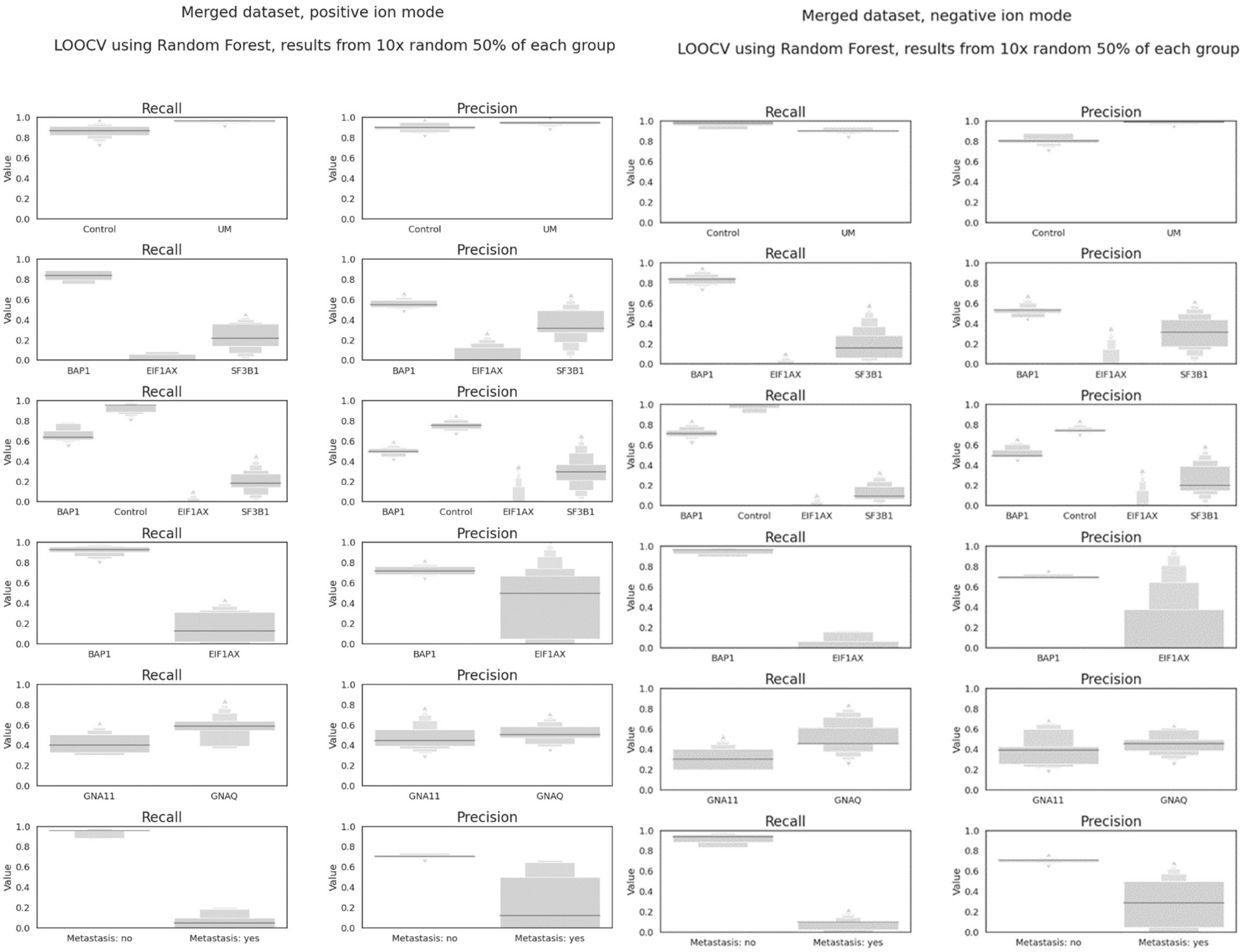


**Figure S9. Repeated leave-one-out cross-validation on random forest classifier.**

Leave-one-out cross-validation (LOOCV) using a random forest classifier trained on 10 bootstraps each selecting a random 50% of the samples per group. Note, that we balanced each (boostrapped) dataset by oversampling each group (i.e. by randomly selecting 200 times a sample from each group with replacement) and addition of normal noise N(0,0.25). The boxplots represent recall and precision per group based on the results of the 10 bootstraps. The merged dataset is used with positive (left panel) and negative ion modes (right panel). These results show a good concordance with the original dataset and reproduce the random forest classifier results in the original experiment.

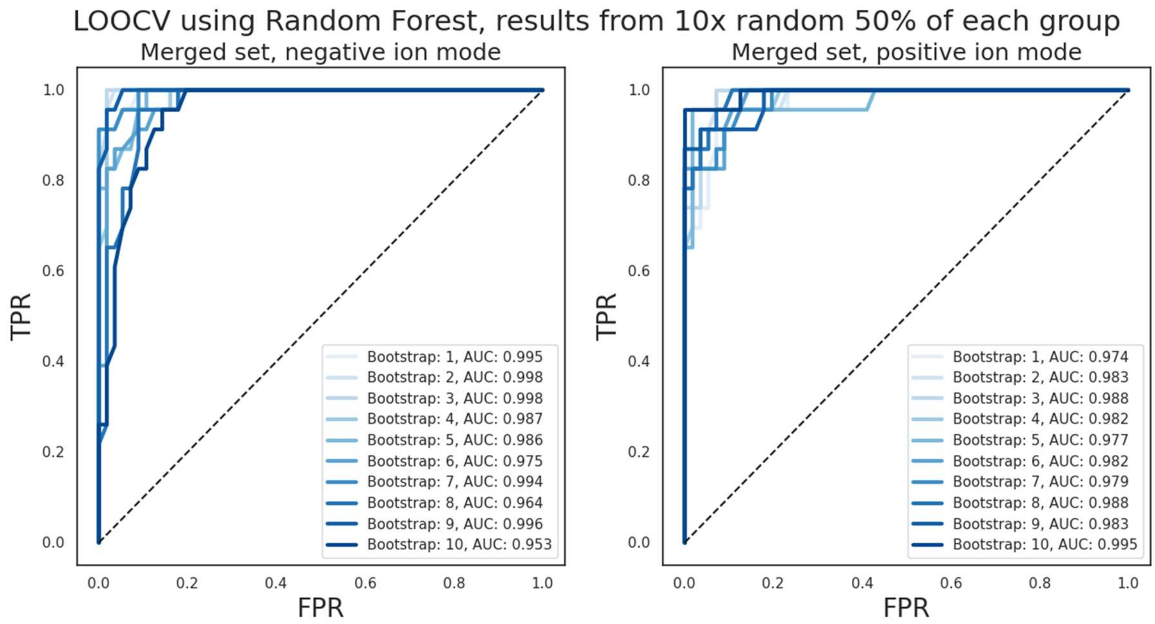


**Figure S10. Receiver operating characteristic of the repeated leave-one-out cross-validation of the random forest classifiers.**

Leave-one-out cross-validation (LOOCV) using a random forest (RF) classifier trained 10 bootstraps on 50% of the sample per group. Each receiver operating characteristic (ROC) curve is based on the probability scores from the RF classifier for one bootstrap, i.e., a random 50% of the samples from the UM and controls samples is selected, where after a LOOCV strategy is deployed.

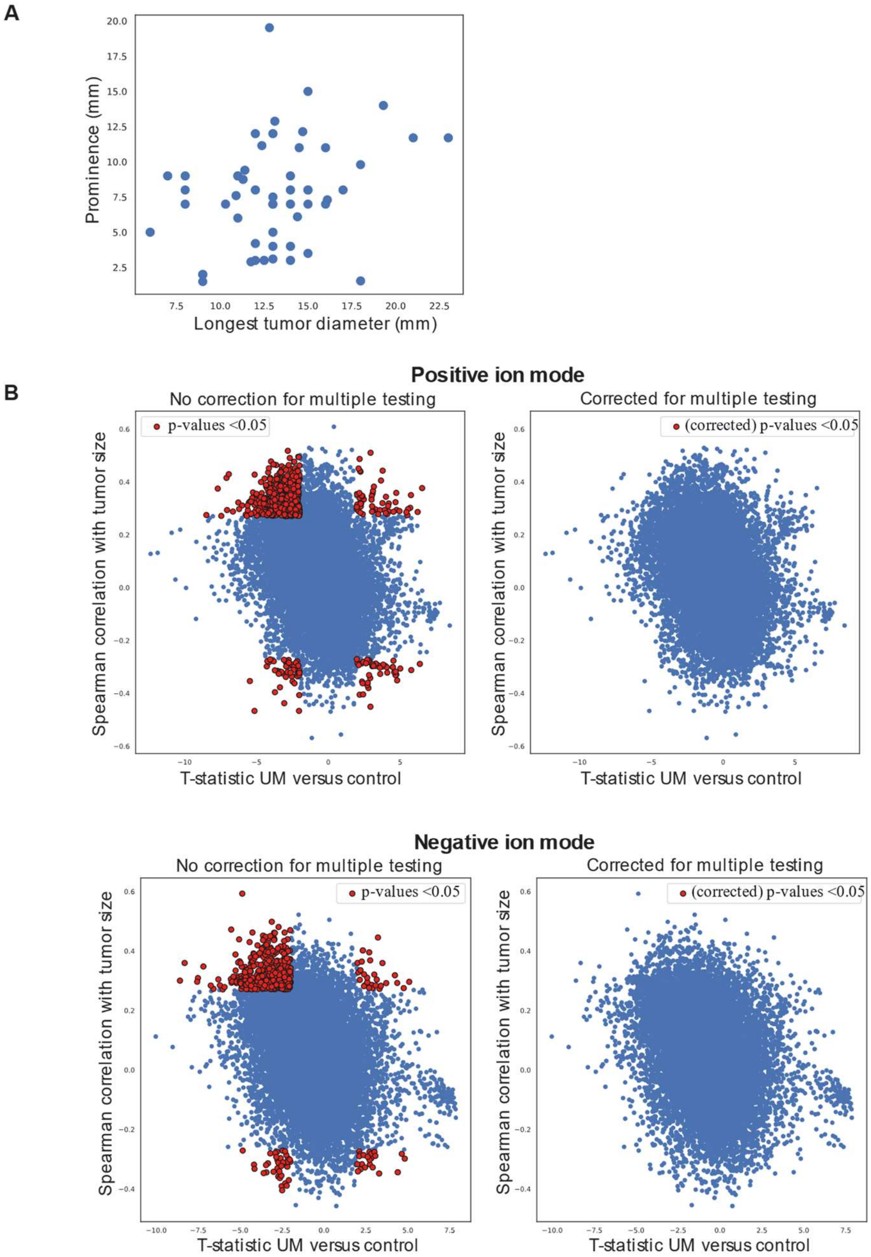


**Figure S11. Correlation of m/z features with tumor size and differential abundance between UM patients and controls.**

Since tumor size characteristics prominence and longest tumor diameter (LTD) correlate, the first PC from PCA analysis was used as metric to correlate with feature abundance (**A**). Next, the t-statistic of feature abundance in UM-patients versus control-participants was calculated. By scattering both metrics, we obtain interesting regions in each corner of the scatter plot and these features are depicted by red dots (p-value <0.05) (**B**). In the top-right corner of the plot the relatively high feature abundance in UM-patients and (strong) correlation with tumor size are depicted; the bottom-left corner shows low feature abundance in UM-patients and inverse correlation to tumor size. No feature is significantly differentially abundant between UM-patients and control-participants and correlated with tumor size, after correcting for multiple testing using the Benjamini/Hochberg procedure with a family-wise error rate of 0.05.

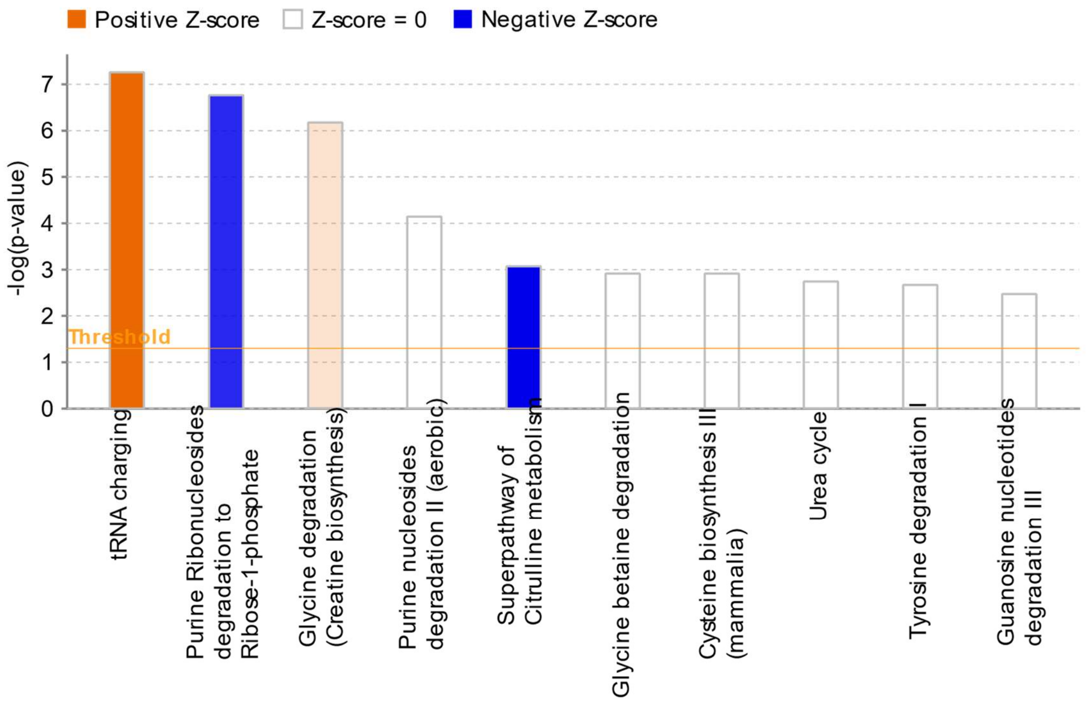


**Figure S12. Pathway analysis of top-10 affected pathways in UM-patients compared to controls.**

All metabolite annotations with a HMDB ID were pulled from both ion modes. For metabolites that were annotated in both ion modes, we selected the ion mode that had on average the highest abundancy (i.e., better signal-to-noise ratio). The fold-change between UM-patients and control-participants and the p-values from the t-test were used as input for Ingenuity Pathway Analysis (IPA). Metabolites were annotated using an in-house developed database as described previously[2]. Data was analyzed using QIAGEN IPA (QIAGEN Inc., https://digitalinsights.qiagen.com/IPA). Z-scores were obtained by calculating the Fold Change between annotated metabolite abundancy in UM-patients compared to control-participants. The p-values were obtained by t-test of differences in metabolite abundancy between UM-patients and control-participants. The differentially abundant annotated metabolites are listed in Table S3. Upregulation of pathways is depicted in orange and downregulation is visualized by blue bars, in which color intensity correlates to the Z-score. We observed an upregulation of transfer RNA (tRNA) charging and the use of glycine for the creatine biosynthesis. Purine ribonucleosides degradation and the super pathway of citrulline metabolism were downregulated in UM-patients.

**Table S1. Characteristics of controls.**
Control-participants are included from the Combined Ophthalmic Research Rotterdam Biobank (CORRBI). These patients had blood drawn prior to surgical treatment and no systemic treatment was administered during the period of phlebotomy. Abbreviation: AMD: Age-related macular degeneration.

| Study ID | Sex | Age at phlebotomy (range) | Disease |
|---|---|---|---|
| **11603** | Male | 76-80 | AMD |
| **11621** | Male | 76-80 | AMD |
| **11628** | Male | 71-75 | Cataract |
| **11643** | Female | 61-65 | AMD |
| **11652** | Female | 76-80 | AMD |
| **11657** | Female | 61-65 | Cataract |
| **11687** | Female | 86-90 | Branch retinal vein occlusion |
| **11743** | Female | 81-85 | AMD |
| **11794** | Male | 81-85 | Traumatic cataract |
| **11819** | Female | 56-60 | Open angle glaucoma |
| **11825** | Male | 61-65 | AMD |
| **11826** | Male | 76-80 | AMD |
| **11828** | Female | 86-90 | AMD |
| **11900** | Female | 61-65 | Cataract |
| **11901** | Female | 61-65 | Cataract |
| **11932** | Male | 81-85 | AMD |
| **11952** | Male | 71-75 | Cataract |
| **11963** | Female | 65-70 | Cataract |
| **11965** | Male | 81-85 | Cataract |
| **11966** | Male | 81-85 | Cataract |
| **11967** | Male | 96-100 | AMD |
| **11969** | Male | 81-85 | Diabetic macular edema |
| **11970** | Female | 56-60 | Cataract |
| **11971** | Male | 66-70 | Cataract |
| **11972** | Male | 81-85 | Cataract |
| **11978** | Female | 76-80 | AMD |
| **12156** | Male | 65-70 | AMD |
| **12157** | Male | 65-70 | Cataract |
| **12158** | Female | 71-75 | Cataract |
| **12162** | Female | 61-65 | Cataract |
| **12163** | Female | 66-70 | Cataract |
| **12165** | Female | 81-85 | AMD |
| **12166** | Male | 71-75 | Cataract |
| **12167** | Male | 61-65 | Cataract |
| **12168** | Female | 65 | AMD |
| **12175** | Female | 88 | AMD |
| **12178** | Female | 74 | AMD |
| **12237** | Male | 76 | Cataract |
| **12280** | Female | 63 | Cataract |
| **12287** | Female | 87 | AMD |
| **12288** | Female | 81 | AMD |
| **12291** | Female | 77 | AMD |
| **12292** | Male | 78 | Cataract |
| **12293** | Female | 75 | Central retinal vein occlusion |
| **12294** | Male | 72 | AMD |
| **12296** | Female | 67 | Cataract |

**Table S2. Patient and sample characteristics of the technical and biological replication sets.** To compare metabolite profiles and evaluate batch-effects a technical replication set comprising of 15 samples was analyzed in both the discovery and replication set. Additionally, three samples were used as a biological replicate in which a different plasma sample was retrieved at the same time-point. The patients resemble their respective cohorts in metastatic free survival (MFS), age at onset and male/female ratio. These samples were retrieved and subsequently stored in -80 °C between 1998 and 2019.

| Study_ID | Sex | Age at onset (years) | DFS (months) | Plasma retrieval | Mutation | Replication set |
|---|---|---|---|---|---|---|
| **EOM-0147** | Male | 56,4 | 95,7 | 1998 | *SF3B1* | Technical replication |
| **EOM-0296** | Male | 66,6 | 8,2 | 2003 | *BAP1* | Technical replication |
| **EOM-0334** | Male | 65,4 | 23,7 | 2004 | *BAP1* | Technical replication |
| **EOM-0355a** | Female | 72,9 | 19,4 | 2004 | *EIF1AX* | Technical replication |
| **EOM-0355** | Female | 72,9 | 19,4 | 2004 | *EIF1AX* | Biological replication |
| **EOM-0409** | Female | 27,3 | 154,1 | 2006 | *SF3B1* | Technical replication |
| **EOM-0464** | Female | 73,5 | 130,9 | 2008 | *SF3B1* | Technical replication |
| **EOM-0472** | Female | 66 | 143,8 | 2008 | *EIF1AX* | Technical replication |
| **EOM-0485** | Male | 82,8 | 63,2 | 2008 | *EIF1AX* | Technical replication |
| **EOM-0501** | Female | 57,5 | 15,8 | 2009 | *BAP1* | Technical replication |
| **EOM-0534** | Male | 66,2 | 48,5 | 2010 | *BAP1* | Technical replication |
| **EOM-0627** | Female | 71,5 | 102,3 | 2011 | *EIF1AX* | Technical replication |
| **EOM-0724** | Female | 43 | 67,5 | 2014 | *SF3B1* | Technical replication |
| **EOM-0779a** | Male | 47,7 | 70 | 2015 | *SF3B1* | Technical replication |
| **EOM-0779** | Male | 47,7 | 70 | 2015 | *SF3B1* | Biological replication |
| **EOM-1097a** | Female | 46 | 11,3 | 2018 | *SF3B1* | Technical replication |
| **EOM-1097** | Female | 46 | 11,3 | 2018 | *SF3B1* | Biological replication |
| **EOM-1104** | Male | 59,9 | 14,3 | 2019 | *EIF1AX* | Technical replication |

Supplementary materials references

1. Bongaerts, M.; Bonte, R.; Demirdas, S.; Jacobs, E.H.; Oussoren, E.; van der Ploeg, A.T.; Wagenmakers, M.; Hofstra, R.M.W.; Blom, H.J.; Reinders, M.J.T.; et al. Using Out-of-Batch Reference Populations to Improve Untargeted Metabolomics for Screening Inborn Errors of Metabolism. *Metabolites* **2020**, *11*, doi:10.3390/metabo11010008.
2. Bonte, R.; Bongaerts, M.; Demirdas, S.; Langendonk, J.G.; Huidekoper, H.H.; Williams, M.; Onkenhout, W.; Jacobs, E.H.; Blom, H.J.; Ruijter, G.J.G. Untargeted Metabolomics-Based Screening Method for Inborn Errors of Metabolism using Semi-Automatic Sample Preparation with an UHPLC- Orbitrap-MS Platform. *Metabolites* **2019**, *9*, doi:10.3390/metabo9120289.
